# MTHFR Polymorphism Is Associated With Severe Methotrexate-Induced Toxicity in Osteosarcoma Treatment

**DOI:** 10.3389/fonc.2021.781386

**Published:** 2021-12-15

**Authors:** Wenchao Zhang, Zhongyue Liu, Zhimin Yang, Chengyao Feng, Xiaowen Zhou, Chao Tu, Zhihong Li

**Affiliations:** ^1^ Department of Orthopaedics, The Second Xiangya Hospital, Central South University, Changsha, China; ^2^ Hunan Key Laboratory of Tumor Models and Individualized Medicine, The Second Xiangya Hospital, Central South University, Changsha, China; ^3^ Xiangya School of Medicine, Central South University, Changsha, China

**Keywords:** MTHFR, polymorphism, osteosarcoma, methotrexate, toxicity

## Abstract

**Background:**

Previous studies have revealed the critical role of methylene tetrahydrofolate reductase (MTHFR) polymorphisms in response to high-dose methotrexate (MTX)-induced toxicity in osteosarcoma patients. However, the conclusions remain controversial. In this setting, we performed a meta-analysis to determine their association more precisely.

**Method:**

Eligible studies were searched and screened in PubMed, Web of Science, Cochrane Library, Clinical-Trials.gov, Embase, and China National Knowledge Infrastructure (CNKI) following specific inclusion and exclusion criteria. The required information was retrieved and collected for subsequent meta-analysis. Association between MTHFR polymorphism and MTX toxicity was evaluated by odds ratios (ORs).

**Results:**

Seven studies containing 585 patients were enrolled and analyzed in this meta-analysis. Overall, the MTX related grade 3-4 liver toxicity was significantly associated with MTHFR rs1801133 allele (T *vs.* C: OR=1.61, 95%CI=1.07-2.42, P=0.024), homozygote (TT *vs.* CC: OR=2.11, 95%CI=1.06-4.21, P=0.011), and dominant genetic model (TT/TC *vs.* CC: OR=3.15, 95%CI=1.30-7.60, P=0.035) in Asian population. Meanwhile, close associations between MTX mediated grade 3-4 mucositis and MTHFR rs1801133 polymorphism were identified in allele contrast (T *vs.* C: OR=2.28, 95%CI=1.49-3.50, P<0.001), homozygote comparison (TT *vs.* CC: OR=4.07, 95%CI=1.76-9.38, P=0.001), heterozygote comparison (TC *vs.* CC: OR=2.55, 95%CI=1.20-5.42, P=0.015), recessive genetic model (TT *vs.* TC/CC: OR=2.09, 95%CI=1.19-3.67, P=0.010), and dominant genetic model (TT/TC *vs.* CC: OR=2.97, 95%CI=1.48-5.96, P=0.002). Additionally, kidney toxicity was corelated with the heterozygote comparison (TC *vs.* CC: OR=2.63, 95%CI=1.31-5.29, P=0.007) of rs1801133 polymorphism.

**Conclusion:**

The MTHFR rs1801133 polymorphism was significantly associated with severer liver toxicity induced by high-dose MTX treatment in the Asian population. In the meantime, patients with MTHFR rs1801133 polymorphism were predisposed to MTX- related mucositis.

## Introduction

Primarily occurring in adolescents, osteosarcoma has been the second malignancy among young teenagers (1) and the most prevalent primary osseous tumor with an annual incidence of 1~3 cases per million worldwide (2). It is characterized by the production of osteoid tissue and immature bone mainly in the metaphysis of long bones (3). Though the prognosis of osteosarcoma has improved significantly over the past decades, outcomes for most patients remain variable, under the influence of multiple elements, for instance, the genetic and epigenetic background (4–7).

The existing treatment for osteosarcoma involves neoadjuvant chemotherapy, lesion resection, and chemotherapy. Adjuvant or neoadjuvant chemotherapy has substantially improved the long-term survival rate since the early 1970s (8). The common chemotherapy regimens comprise high-dose methotrexate (MTX), doxorubicin, ifosfamide, cisplatin, and vincristine. However, the chemotherapy-related toxicity and adverse effects remain intractable and unpredictable, which are the main obstacles that lead to dose decrease and even interruption or discontinuation of chemotherapy. MTX, an inhibitor of dihydrofolate reductase, plays a role in interrupting the DNA synthesis and normal cellular metabolism in both the cancerous and normal cells (9). Previous studies have reported a high incidence of medication toxicity during high-dose MTX treatment for osteosarcoma patients (10, 11). Adverse events induced by high-dose MTX (>1g/m^2^) include renal insufficiency, hepatocellular damage, nausea/vomiting, skin/subcutaneous induration, anemia, mucositis, etc. (10). The presence of toxicity is influenced by multiple factors such as age, gender, ethnicity, and genetic background (9, 12). In this setting, patients may benefit from individualized chemotherapy that is tailored according to their disease characteristics and background.

Methylene tetrahydrofolate reductase (MTHFR) is a crucial enzyme in the folate metabolism and DNA synthesis regulatory network, which promotes the conversion of 5,10-methylenetetrahydrofolate to 5-methyltetrahydrofolate (13). Until now, two types of polymorphisms have been identified for MTHFR, containing rs1801133 and rs1801131. Rs1801133 polymorphism is characterized by the C to T substitution at nucleotide position 677, leading to amino acid change from alanine to valine, which decreases the enzymatic activity significantly by more than 30% (14). While nucleotide 1289 A is substituted with C in rs1801131. Studies have shed light on the relationship between different MTHFR variants and MTX treatment toxicity in various diseases. For instance, Lv et al. have shown more frequent MTX-related side effects in MTHFR-TT carriers compared with MTHFR-677CC in rheumatoid arthritis (RA) patients (15). The evidence also indicated that the MTHFR 677T mutation decreased the chemosensitivity of breast cancer cells to MTX (16). And the MTHFR C677T polymorphism was remarkably associated with relapse after MTX treatment in pediatric acute lymphoblastic leukemia (ALL) (17). Meanwhile, several studies have revealed the association between MTHFR variants and MTX toxicity in osteosarcoma (18–24). However, the current conclusions remain controversial. Therefore, we conducted this meta-analysis, with the aim to reach a more precise consensus.

## Materials and Methods

### Search Strategy

This meta-analysis follows the instruction of Preferred Reporting Items for Systematic Reviews and Meta-Analyses (PRISMA) guidelines (25). Studies related to the meta-analysis topic were retrieved from PubMed, Web of Science, Cochrane Library, Clinical-Trials.gov, Embase, and China National Knowledge Infrastructure (CNKI) under the search terms “MTHFR and (polymorphism or variant or mutation) and osteosarcoma” updated on July 26, 2021. Two researchers (WCZ, ZYL) screened and selected the eligible studies independently in all the research hits by reviewing their title, abstract or full text.

### Inclusion and Exclusion Criteria

All enrolled studies were sorted according to the specific inclusion and exclusion criteria. The inclusion criteria include: (1) the case control study, (2) assessment of the association between MTHFR polymorphism and MTX toxicity in the treatment of osteosarcoma, (3) containing available allele and genotype distribution information to calculate odds ratios (ORs) and 95% confidence interval (CI). Accordingly, the exclusion criteria were: (1) studies with duplicate data, (2) articles such as conference abstracts, letters, reviews, case reports, sequencing data, bioinformatic analyses, and meta-analyses, (3) studies without extractable toxicity response grouped by detailed genotyping.

### Data extraction and Quality Evaluation

Two independent researchers (WCZ, ZYL) extracted all needed information from the included studies, comprising the first author’s name, published year, country, ethnicity, genotyping methods, sample size, investigated SNPs, MTX dose, ORs, 95% CI for different genotype (allele contrast T vs. C, homozygote comparison TT vs. CC, heterozygote comparison TC vs. CC, recessive genetic model TT vs. TC/CC, and dominant genetic model TT/TC vs. CC), and MTX related toxicity (liver toxicity, kidney toxicity, mucositis, and anemia). The result was then checked and confirmed by another researcher (ZMY). The quality of each enrolled study was assessed using the Newcastle-Ottawa Scale (NOS) as previously described (26).

### Statistical Analyses

The ORs and the corresponding 95% CI were calculated to assess the relationship between MTHFR polymorphisms and MTX toxicity. An OR>1 connoted a risk factor for the analyzed outcome, while an OR<1 indicated a protective factor. Four MTX-related adverse events were evaluated under different genotype contrasts. The ORs and 95% CI from all enrolled studies were pooled by using the Stata software (Version 12.0; StataCorp LP, College Station, TX, USA). The fixed model (Mantel-Haenszel method) was used if the heterogeneity was not significant (I^2^<50%, P>0.05), otherwise the random model (DerSimonian and Laird method) was adopted as previously described (27). For several analyses with great heterogeneity, we performed a subgroup analysis to determine the sources of heterogeneity including sample size, genotyping method, and ethnicity.

Meanwhile, the stability of the results was evaluated by sensitivity analysis, which determined the impact of every single study on the pooled results through recalculation after deleting each one. Egger’s linear regression test and Begg’s test were utilized to investigate the potential publication bias. An asymmetric plot indicated the possibility of publication bias. Statistical significance was defined as *p*<0.05 in all statistical analyses.

## Results

### Enrolled Studies and Quality Assessment

**Figure 1:**
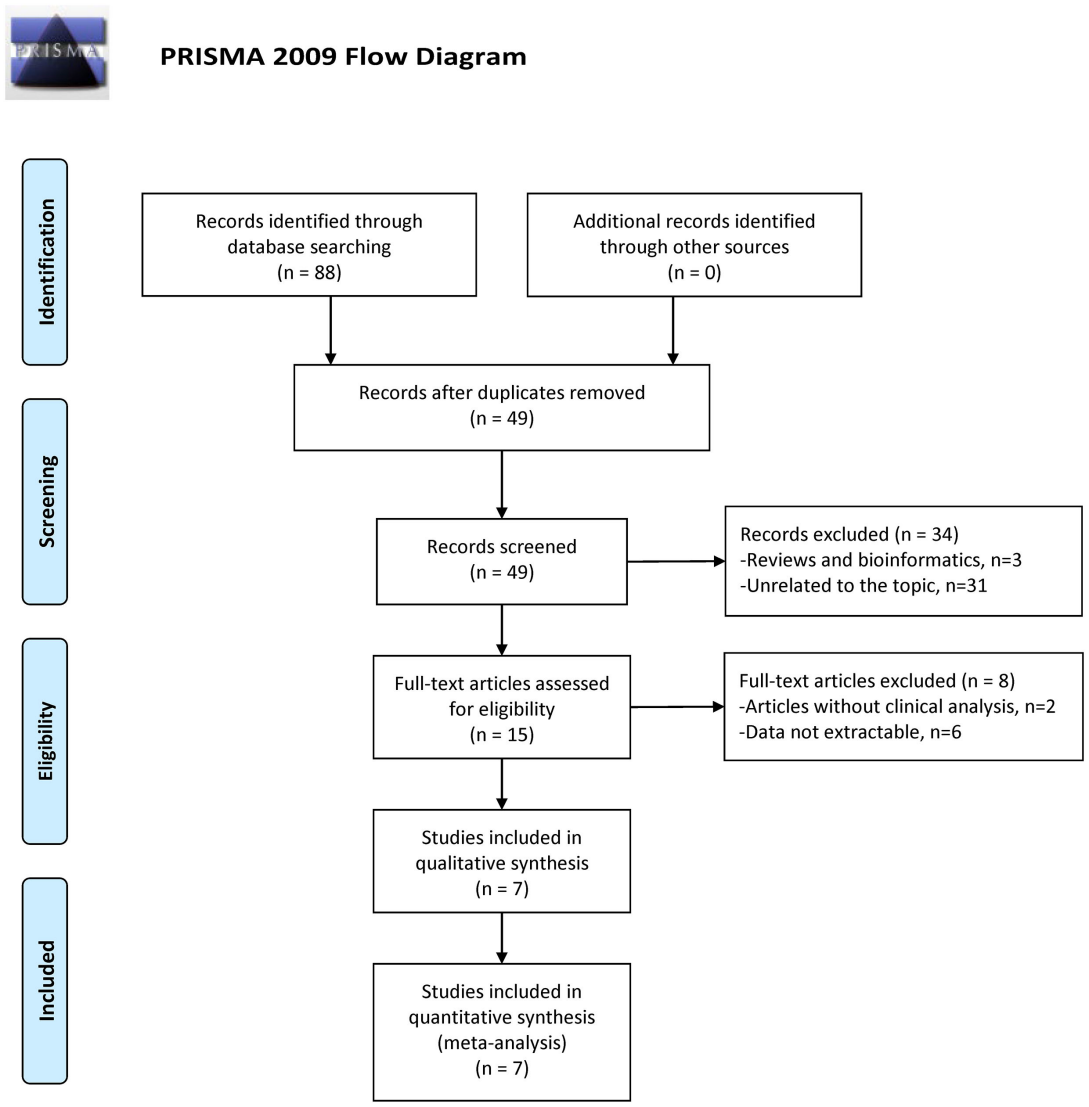
Flow diagram for study identification with criteria in the meta-analysis (28).

Overall, 88 search results were identified in multiple databases according to the search strategy. After duplicates removal, there were 49 studies remaining, and 34 records of reviews, bibliometrics, or unrelated to the topic were further excluded. Subsequently, the full text of 15 studies were screened and 8 studies were obviated because of the lack of extractable clinical data. Finally, seven eligible studies remained for the next step analysis. PRISMA flowchart showed the detailed processes (**Figure 1**). The NOS scores of the included studies were all higher than 7, indicating the adequate quality of these researches.

### Characteristics of Included Studies

Overall, this meta-analysis has included seven studies containing 585 patients through careful screening (**Figure 1**). Four studies focused on the rs1801133 and rs1801131 polymorphisms while three only mentioned the rs1801133. Since data for rs1801131 polymorphism were unextractable in three studies, we can only analyze the association between rs1801133 polymorphism and MTX toxicity. Of all studies, four principal adverse events were construed, comprising liver toxicity, kidney toxicity, mucositis, and anemia. As to the ethnicity, three studies investigated the Caucasian population and four studies focused on the Asian population. Genotyping methods included Microarray, MassARRAY, PCR, and TaqMan SNP Genotyping Assay. The sample size ranged from 37 to 210 with a mean size of 83.57. The MTX dosage was 12 g/m^2^ in most cases while one study administered 200mg/kg of MTX. More specific characteristics of enrolled studies were listed in **Table 1**.

**Table 1:** Characteristics of all enrolled studies.

Author	Year	Country	Ethnicity	Genotyping methods	Sample size	Investigated SNPs	Methotrexate dose	NOS score
Windsor (19)	2012	United Kingdom	Caucasian	Microarray	60	rs1801133, rs1801131	12 g/m^2^	7
Jabeen (20)	2015	Norway	Caucasian	MassARRAY	62	rs1801133	12 g/m^2^ (mean)	8
Park (21)	2016	Korea	Asian	MassARRAY	37	rs1801133, rs1801131	12 g/m^2^	7
Lambrecht (22)	2017	Belgium	Caucasian	PCR	48	rs1801133	12 g/m^2^	8
Xie (23)	2018	China	Asian	RT-PCR	59	rs1801133, rs1801131	10-12 g/m^2^ week twice	8
Xu (24)	2018	China	Asian	TaqMan SNP Genotyping Assay	109	rs1801133, rs1801131	10 g/m^2^/d	8
Ren (29)	2011	China	Asian	RT-PCR	210	rs1801133	200mg/kg	7

### Quantitative Synthesis Revealing Toxicity Related Genotype

**Figure 2:**
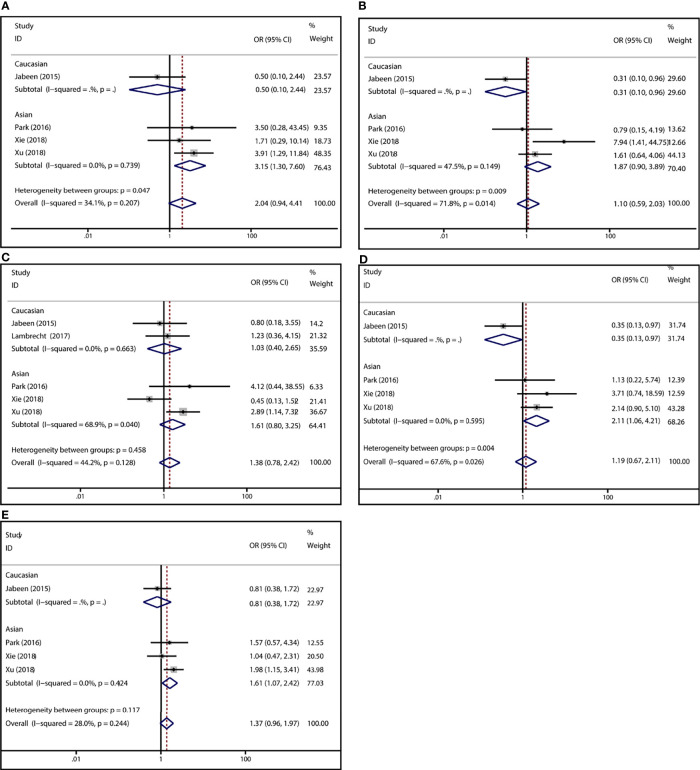
Forest plots for the association between MTX-induced liver toxicity and MTHFR rs1801133 polymorphism. **(A)** homozygote comparison TT vs. CC; **(B)** heterozygote comparison TC vs. CC; **(C)** recessive genetic model TT vs. TC/CC; **(D)** dominant genetic model TT/TC vs. CC; **(E)** allele contrast T vs. C.

**Figure 3:**
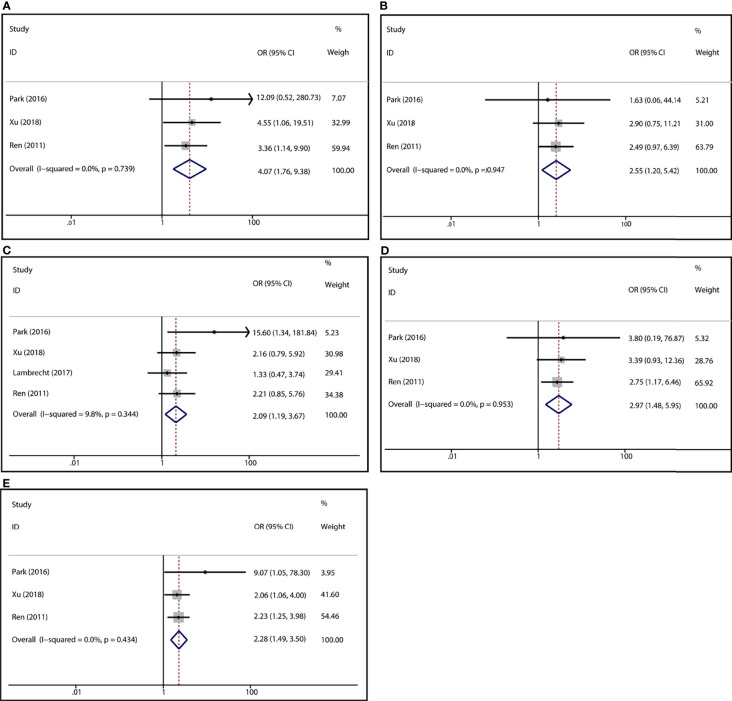
Forest plots for the association between MTX-induced mucositis and MTHFR rs1801133 polymorphism. **(A)** homozygote comparison TT vs. CC; **(B)** heterozygote comparison TC vs. CC; **(C)** recessive genetic model TT vs. TC/CC; **(D)** dominant genetic model TT/TC vs. CC; **(E)** allele contrast T vs. C.

**Figure 4:**
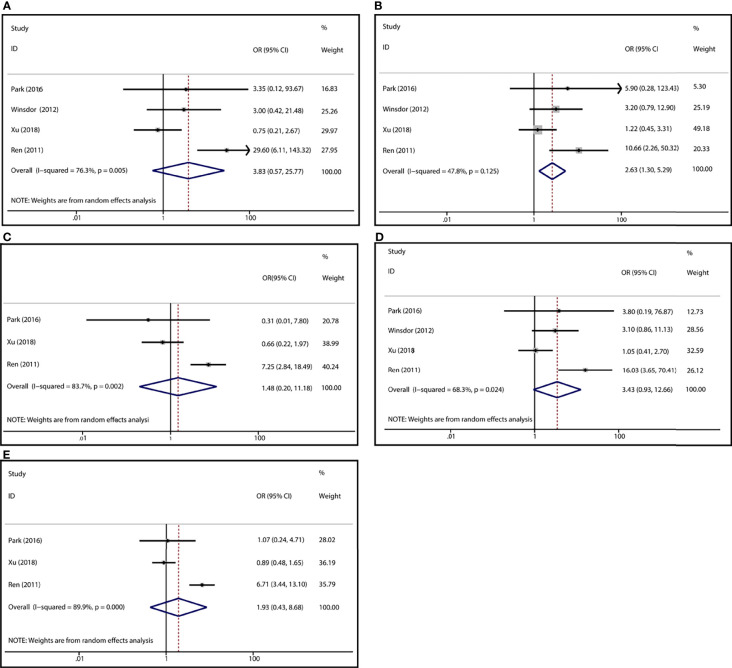
Forest plots for the association between MTX-induced Kidney toxicity and MTHFR rs1801133 polymorphism. **(A)** homozygote comparison TT vs. CC; **(B)** heterozygote comparison TC vs. CC; **(C)** recessive genetic model TT vs. TC/CC; **(D)** dominant genetic model TT/TC vs. CC; **(E)** allele contrast T vs. C.

**Figure 5:**
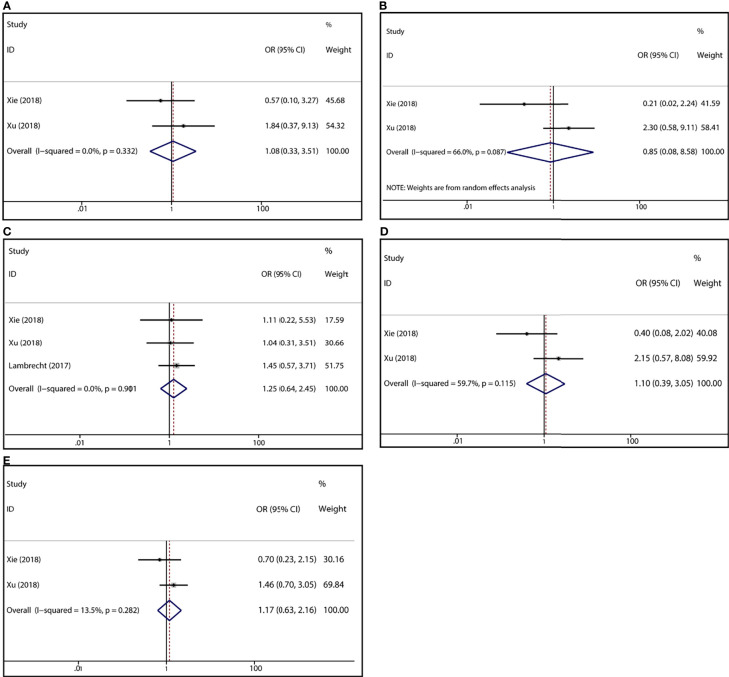
Forest plots for the association between MTX-induced Anemia and MTHFR rs1801133 polymorphism. **(A)** homozygote comparison TT vs. CC; **(B)** heterozygote comparison TC vs. CC; **(C)** recessive genetic model TT vs. TC/CC; **(D)** dominant genetic model TT/TC vs. CC; **(E)** allele contrast T vs. C.

To demonstrate the association between MTX toxicity and various genotype, ORs and 95% CI from different studies were incorporated (data were shown in **Table 2**). Summarily, the MTX-related high-level liver toxicity (grade 3–4) was significantly associated with MTHFR rs1801133 polymorphism under allele contrast (T vs. C: OR=1.61, 95%CI=1.07-2.42, P=0.024), homozygote comparison (TT vs. CC: OR=2.11, 95%CI=1.06-4.21, P=0.011), and dominant genetic model (TT/TC vs. CC: OR=3.15, 95%CI=1.30-7.60, P=0.035) in the Asian population but not in the overall population (**Figure 2**). Meanwhile, close relations between MTX mediated high level mucositis (grade 3–4) and MTHFR rs1801133 polymorphism were identified in allele contrast (T vs. C: OR=2.28, 95%CI=1.49-3.50, P<0.001), homozygote comparison (TT vs. CC: OR=4.07, 95%CI=1.76-9.38, P=0.001), heterozygote comparison (TC vs. CC: OR=2.55, 95%CI=1.20-5.42, P=0.015), recessive genetic model (TT vs. TC/CC: OR=2.09, 95%CI=1.19-3.67, P=0.010), and dominant genetic model (TT/TC vs. CC: OR=2.97, 95%CI=1.48-5.96, P=0.002) (**Figure 3**). Additionally, the presence of the TC genotype indicated a high risk of kidney toxicity compared to the CC genotype (TC vs. CC: OR=2.63, 95%CI=1.31-5.29, P=0.007) (**Figure 4**). There was no correlation between rs1801133 polymorphism and MTX-related Anemia (**Figure 5**).

**Table 2:** Meta-analysis of the MTHFR polymorphisms with MTX-related toxicity.

Comparison	Ethnicity	N	OR	Low 95%CI	High 95%CI	P	Mode	Heterogeneity			Sensitive analysis	Publication bias	
								χ^2^	P	I^2^		Begg’s Test p-value	Egger’s test p-value
Liver toxicity													
TT vs CC	Caucasian	1	0.81	0.38	1.71	NA	NA	NA	NA	NA	NA	1.000	0.697
	Asian	3	3.15	1.30	7.60	0.011	Fixed	0.61	0.739	0.0%	Good		
	Overall	4	2.04	0.94	4.41	0.218	Fixed	4.55	0.207	34.1%	Good		
TC vs CC	Caucasian	1	0.31	0.10	0.96	NA	NA	NA	NA	NA	NA	0.734	0.718
	Asian	3	1.87	0.90	3.89	0.095	Fixed	3.81	0.149	47.5%	Good		
	Overall	4	1.23	0.36	4.20	0.746	Random	10.64	0.014	71.8%	Good		
TT vs. TC/CC	Caucasian	2	1.03	0.40	2.65	0.945	Fixed	0.19	0.663	0.0%	Good	1.000	0.856
	Asian	3	1.58	0.39	6.45	0.521	Random	6.42	0.04	68.9%	Good		
	Overall	5	1.38	0.78	2.42	0.265	Fixed	7.16	0.128	44.2%	Good		
TT/TC vs. CC	Caucasian	1	0.35	0.13	0.97	NA	NA	NA	NA	NA	NA	0.734	0.836
	Asian	3	2.11	1.06	4.21	0.043	Fixed	1.04	0.595	0.0%	Good		
	Overall	4	1.26	0.43	3.67	0.035	Random	9.25	0.024	67.6%	Good		
T vs C	Caucasian	1	0.81	0.38	1.72	NA	NA	NA	NA	NA	NA	1.000	0.477
	Asian	3	1.61	1.07	2.42	0.024	Fixed	1.72	0.424	0.0%	Good		
	Overall	4	1.37	0.96	1.97	0.085	Fixed	4.17	0.244	28.0%	Good		
Kidney toxicity													
TT vs CC	Overall	4	3.82	0.57	25.78	0.168	Random	12.66	0.005	76.3%	Good	0.734	0.748
TC vs CC	Overall	4	2.63	1.31	5.29	0.007	Fixed	5.57	0.125	47.8%	Good	0.308	0.340
TT vs. TC/CC	Overall	3	1.48	0.20	11.18	0.704	Random	12.23	0.002	83.7%	Good	1.000	0.659
TT/TC vs. CC	Overall	4	3.43	0.93	12.66	0.064	Random	9.46	0.024	68.3%	Good	0.734	0.465
T vs C	Overall	3	1.93	0.43	8.67	0.392	Random	19.87	0.000	89.9%	Good	1.000	0.936
Mucositis													
TT vs CC	Overall	3	4.07	1.76	9.38	0.001	Fixed	0.60	0.739	0.0%	Good	0.296	0.063
TC vs CC	Overall	3	2.55	1.20	5.42	0.015	Fixed	0.11	0.947	0.0%	Good	1.000	0.603
TT vs. TC/CC	Overall	4	2.09	1.19	3.67	0.010	Fixed	3.33	0.344	9.8%	Good	1.000	0.134
TT/TC vs. CC	Overall	3	2.97	1.48	5.96	0.002	Fixed	0.10	0.953	0.0%	Good	1.000	0.385
T vs C	Overall	3	2.28	1.49	3.50	0.000	Fixed	1.67	0.434	0.0%	Good	1.000	0.173
Anemia													
TT vs CC	Overall	2	1.08	0.33	3.51	0.092	Fixed	0.94	0.322	0.0%	NA	NA	NA
TC vs CC	Overall	2	1.26	0.38	4.13	0.890	Random	2.94	0.087	66.0%	NA	NA	NA
TT vs. TC/CC	Overall	3	1.25	0.64	2.45	0.521	Fixed	0.21	0.901	0.0%	Good	1.000	0.441
TT/TC vs. CC	Overall	2	1.10	0.39	3.05	0.992	Random	2.48	0.115	59.7%	NA	NA	NA
T vs C	Overall	2	1.17	0.63	2.16	0.617	Fixed	1.16	0.282	13.5%	NA	NA	NA

NA, Not available.

### Heterogeneity Analysis

Significant heterogeneity has been identified in rs1801133 polymorphism and liver toxicity. Considering the potential sources of heterogeneity including ethnicity, genotyping method, and sample size, subgroup analysis uncovered that grouping by ethnicity obviously decreased the initial heterogeneity. Meanwhile, remarkable heterogeneity existed in rs1801133 polymorphism and kidney toxicity. Meta-regression was unable to identify the potential source of heterogeneity among various factors containing publication year, ethnicity, genotyping method, and sample size. However, the elimination of one study by Ren et al. could substantially reduce the heterogeneity.

### Sensitive Analysis

**Figure 6:**
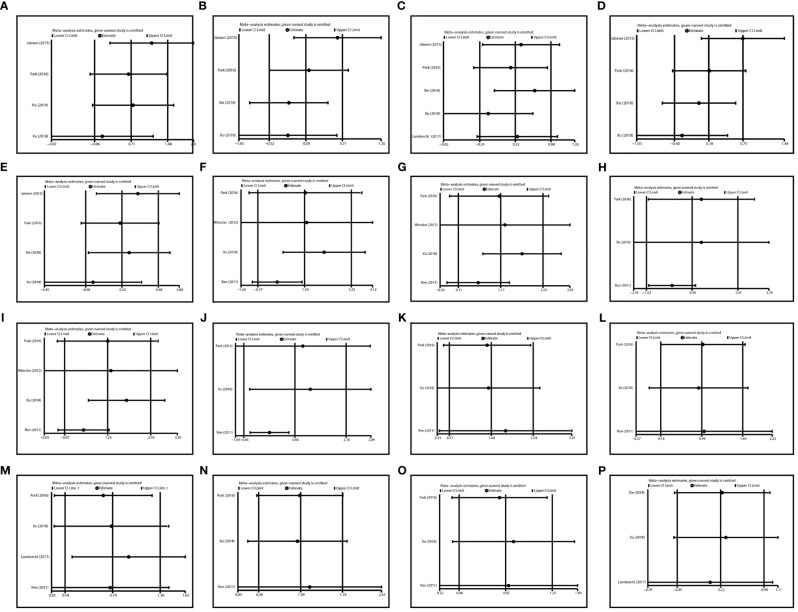
Sensitivity analysis in various comparisons. **(A)** TT vs CC in liver toxicity; **(B)** TC vs CC in liver toxicity; **(C)** TT vs. TC/CC in liver toxicity; **(D)** TT/TC vs. CC in liver toxicity; **(E)** T vs C in liver toxicity; **(F)** TT vs CC in kidney toxicity; **(G)** TC vs CC in kidney toxicity; **(H)** TT vs. TC/CC in kidney toxicity; **(I)** TT/TC vs. CC in kidney toxicity; **(J)** T vs. C in kidney toxicity; **(K)** TT vs CC in Mucositis; **(L)** TC vs CC in Mucositis; **(M)** TT vs. TC/CC in Mucositis; **(N)** TT/TC vs. CC in Mucositis; **(O)** T vs. C in Mucositis; **(P)** TT vs. TC/CC in Anemia.

Sensitive analysis was performed by recalculating the pooled ORs and 95% CI after dislodging each individual study. The removal of any single study did not affect the quantitative results significantly (**Table 2**, **Figure 6**), suggesting the reliability of this analysis.

### Publication Bias

**Figure 7:**
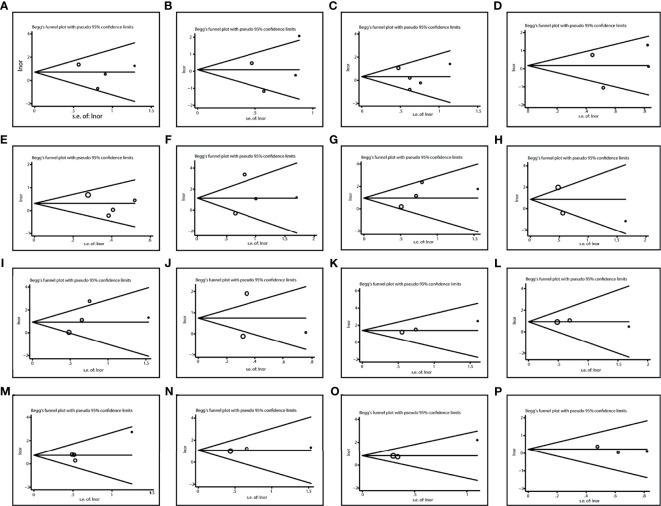
Publication bias in various comparisons. **(A)** TT vs CC in liver toxicity; **(B)** TC vs CC in liver toxicity; **(C)** TT vs. TC/CC in liver toxicity; **(D)** TT/TC vs. CC in liver toxicity; **(E)** T vs C in liver toxicity; **(F)** TT vs CC in kidney toxicity; **(G)** TC vs CC in kidney toxicity; **(H)** TT vs. TC/CC in kidney toxicity; **(I)** TT/TC vs. CC in kidney toxicity; **(J)** T vs. C in kidney toxicity; **(K)** TT vs CC in Mucositis; **(L)** TC vs CC in Mucositis; **(M)** TT vs. TC/CC in Mucositis; **(N)** TT/TC vs. CC in Mucositis; **(O)** T vs. C in Mucositis; **(P)** TT vs. TC/CC in Anemia.

We used Egger’s test and Begg’s test to identify potential publication bias among studies. No evidence of publication bias was found (**Table 2**). And the Begg’s test funnel plots did not show obvious asymmetry (**Figure 7**).

## Discussion

Treatment for osteosarcoma has made substantial progress since the adoption of several effective therapeutic strategies over the past decades, including but not limited to adjuvant or neoadjuvant chemotherapy (30). The backbone for treatment comprises the MTX, cisplatin, doxorubicin, and ifosfamide, which have shown great efficacy in osteosarcoma management (31). However, chemotherapy-related toxicities have contributed to a variety of adverse outcomes, varying highly among patients. In most cases, patients are stratified largely relying on their concrete characteristics such as the clinical manifestations, radiographic features, pathological biopsy, etc. And they are prescribed with a relatively fixed regimen schedule even in those with or without metastases at diagnosis (32), leading to unsatisfied outcome. Fortunately, the biological biomarkers especially genome feature may conduce to more precise stratification and therapeutic optimization. Of the current studies, impact of pharmacogenetics on drug toxicities in osteosarcoma have been largely focused on (33), which includes genes related to DNA repair (34), drug metabolism associated genes (35), and genes involved in drug transport (36).

Although MTX has achieved great clinical success, its unpredictable toxicities such as liver failure, kidney damage, mucositis, hematologic toxicity, anemia, cardiotoxicity, and ototoxicity remain challenging in clinical management, especially in high-dose usage (9, 33). As aforementioned, MTHFR participates in MTX metabolism and its single nucleotide polymorphisms (SNPs) including rs1801133 and rs1801131 may partially determine drug toxicity. Considering the contradictory results among different studies and the insufficient reliability of single study, we have reviewed the existing studies and conducted a meta-analysis to reduce random error.

In this meta-analysis, we have interrogated the relationship between MTHFR rs1801133 polymorphism and MTX-induced toxicities. The findings suggested that grade 3-4 liver toxicity was significantly associated with rs1801133 polymorphism under various contrasts in the Asian population but not in the overall population, indicating the influence of ethnicity on the toxicity-polymorphism association. Previous studies have shown the inconsistences of MTX-related toxicities in populations from different ethnicities (37). Meanwhile, a significant association was also noticed between grade 3-4 mucositis and MTHFR rs1801133 polymorphism. Patients with C to T variants are more vulnerable to MTX-related mucositis, which was similar to a previous study (38). Particularly, a retrospective cohort study in Chinese pediatric patients revealed the close relevance of MTHFR rs1801133 polymorphism to mucositis (39). In other conditions such as RA and hematological malignancies, a close relationship between MTHFR C677T polymorphism and risk of hepatic or gastrointestinal toxicities has also been demonstrated (38, 40, 41). Additionally, high-grade (grade 3-4) kidney toxicity was correlated with the heterozygote comparison (TC vs. CC) of rs1801133 polymorphism but not in other genotype contrasts. This result may be unreliable due to the high heterogeneity among studies. Further, no association was identified between rs1801133 polymorphism and anemia, which was consistent with the finding in hematological malignancies by Zhao et al. (40).

High-dose MTX (>1 g/m^2^) is usually adopted for the treatment of osteosarcoma. An increase in efficacy is accompanied by a high risk of MTX-induced toxicity. Despite the usage of leucovorin rescue to mitigate adverse events, it remains challenging in overcoming severe toxicities in every individual. In this setting, upfront knowledge of drug toxicity based on the patients’ genetic features may pave the way for individualized management and optimization. Herein, we have suggested the close association between MTHFR rs1801133 polymorphism and various MTX toxicities, providing a potential tool to prognosticate the patient’s drug exposure and sensitivity to toxicities.

Although this meta-analysis has interrogated the significant relationship between MTHFR polymorphism and MTX-induced toxicities comprehensively, there are still some limitations. In the first place, the included studies in this meta-analysis investigated the Asian and Caucasian population, but lack the data for other ethnicities such as the African population. Populations from different ethnicities vary in lifestyle and genetic background. Thus, the conclusion may be not representative of all populations. Secondly, only seven studies were included in this meta-analysis, so the sample size is relatively small. Further studies on this topic are needed to enrich the current conclusions, for instance, analysis for another MTHFR polymorphism, rs1801131. Thirdly, heterogeneity in the analysis of kidney toxicity and MTHFR polymorphism was significant. However, the source of heterogeneity was untrackable because of the limited data. Fourthly, this meta-analysis was limited by the insufficient available data, thus factors regarding age, gender, surgery, radiation, etc. could not be analyzed to reach a more comprehensive conclusion.

## Conclusion

To date, this is the first meta-analysis in regard to the association between MTHFR polymorphism and MTX-induced toxicities. A significant relationship between the rs1801133 variant and MTX-related hepatic toxicity in the Asian population has been identified. Meanwhile, mucositis was closely correlated with the rs1801133 polymorphism under various comparisons. In clinical implementations, genotyping patients according to their MTHFR polymorphism for tailored treatment largely contributes to the enhancement of treatment outcomes in osteosarcoma.

## Data Availability Statement

The original contributions presented in the study are included in the article/supplementary material. Further inquiries can be directed to the corresponding authors.

## Author Contributions

WZ and ZYL conceived and designed the work. Material preparation, data collection and analysis were performed by WZ, ZY, and ZYL. The first draft of the manuscript was written by WZ. ZYL, CF, and XZ wrote sections of the manuscript. All authors commented on previous versions of the manuscript. CT revised the manuscript. ZHL contributed to article drafting, critical revision and final approval of the version to be published. All authors contributed to the article and approved the submitted version.

## Funding

This work was funded by the National Natural Science Foundation of China (No. 81902745, No.82172500, No.82103228), Hunan Provincial Research and Development Program in Key Areas (2020DK2003), and China Postdoctoral Science Foundation (No. 2021M693557).

## Conflict of Interest

The authors declare that the research was conducted in the absence of any commercial or financial relationships that could be construed as a potential conflict of interest.

## Publisher’s Note

All claims expressed in this article are solely those of the authors and do not necessarily represent those of their affiliated organizations, or those of the publisher, the editors and the reviewers. Any product that may be evaluated in this article, or claim that may be made by its manufacturer, is not guaranteed or endorsed by the publisher.
